# 亲水作用液相色谱脱除人参提取物中农药残留

**DOI:** 10.3724/SP.J.1123.2020.08017

**Published:** 2021-04-08

**Authors:** Lingli SUN, Jia LIU, Xiujie GUO, Lidong WU, Zhengchao DUAN, Chaoran WANG, Lianzhi WANG

**Affiliations:** 1.湖北民族大学, 湖北 恩施 445000; 1. Hubei Minzu University, Enshi 445000, China; 2.中国科学院大连化学物理研究所, 辽宁 大连 116023; 2. Dalian Institute of Chemical Physics, Chinese Academy of Sciences, Dalian 116023, China; 3.中科院大化所中国医药城生物医药创新研究院, 江苏 泰州 225300; 3. DICP-CMC Innovation Institute of Medicine, Taizhou 225300, China; 4.中国水产科学研究院, 北京 100141; 4. Chinese Academy of Fishery Sciences, Beijing 100141, China

**Keywords:** 亲水作用液相色谱, 人参皂苷, 农药残留, 人参提取物, 脱除技术, hydrophilic interaction liquid chromatography (HILIC), ginsenosides, pesticide residues, ginseng extracts, removal method

## Abstract

亲水作用液相色谱法(HILIC)是一种用于改善强极性物质的保留和分离选择性的方法,广泛应用于药物分析、代谢组学、蛋白质组学等领域。该文利用农药分子与皂苷成分在HILIC上的保留行为差异,开发了一种农药残留脱除方法。以市售高纯人参提取物为例,该文评价了农药分子和人参皂苷在亲水色谱柱上的保留行为,并考察了上样量、淋洗体积、上样体积等因素对农残脱除效果的影响。实验结果证明:7种人参皂苷由于糖链上的羟基与亲水色谱固定相上的羧基形成氢键作用而具有较强保留,而农药分子由于亲水性较差且相对分子质量较小,保留很弱,从而一步实现了7种人参皂苷的富集与14种农残的脱除。在优化所得的最佳脱除工艺条件下,最终制备得到的人参总皂苷样品中,总皂苷的含量由59.87%提高到69.61%;总皂苷的回收率为94.4%;通过气相色谱-三重四极杆质谱(GC-MS/MS)对样品中的农残进行定量检测,发现原人参提取物中14种农残均得到了有效脱除,其中5种含量降至0.05 mg/kg以下,9种完全脱除。本研究是亲水色谱在中药提取物中农残脱除领域的应用,为天然产物的精制提供了一种新的技术手段,该技术对人参提取物中的农残脱除率高、人参总皂苷回收率高且安全、高效、无污染,为高品质人参提取物的研制提供了新的思路。

## Abstract (en)

Ginseng extracts are rich in a variety of ginseng monomer saponins, which have pharmacological functions of retarding aging, enhancing immunity, stimulating blood circulation, and lowering blood pressure. Ginseng is widely used in health products and dietary supplements in the domestic and foreign market. However, the amount of pesticide residues is an important index for measuring the quality of ginseng and ginseng extracts. Therefore, studies focused on methods for the removal of pesticide residues in ginseng extract are of great significance. Hydrophilic interaction liquid chromatography (HILIC) is used to improve the retention and separation selectivity of strongly polar substances, and it is widely employed in drug analysis, metabolomics, proteomics, etc. In this study, a method for the removal of pesticide residues was developed based on the difference in the retention behavior of pesticide residues and ginsenosides on the HILIC column. Using commercially available ginsenoside extracts, the retention behaviors of pesticide residues and ginsenosides on reverse chromatography and hydrophilic chromatographic columns were evaluated by high performance liquid chromatography. The results proved that on the reversed-phase liquid chromatography (RPLC) stationary phase, in addition to the strong retentions of quintozene and pentachloroaniline, which could be clearly separated from the saponins, the retentions of the other five pesticide residues including carbendazim, azoxystrobin, procymidone, iprodione and propiconazole were similar to total ginsenosides. The seven ginsenosides showed strong retention due to the formation of hydrogen bonds between the hydroxyl groups on the sugar chain and the carboxyl groups on the HILIC stationary phase. However, the pesticide residues were not well retained because of their poor hydrophilicity and small molecular weights. For this reason, the pesticide residues and ginsenosides could be completely separated on the HILIC column. Thus, enrichment of the seven ginsenosides and removal of the 14 pesticide residues was realized in one step on the HILIC column. In addition, the effects of loading amount, loading volume, and washing volume on the removal of pesticide residues in ginsenosides were investigated using the Click XIon SPE column. Then, taking the ginsenoside recoveries and pesticide residue removal rates into account, we confirmed the following: the ratio of the maximum sample loading mass to the filler mass was 1∶10; the optimal elution volume was twice the column volume; and the optimal loading volume was twice the column volume. The ginseng extracts were solvated with a 95% ethanol solution and loaded onto an HILIC column. The sample was subjected to pesticide residue removal, and ginsenoside purification and enrichment under the optimum removal conditions. Gradient elution was carried out using ethanol and water as the mobile phases. The total ginsenoside content in the final extracts was increased to 69.61%. The recovery of the total ginsenosides was 94.4%. The pesticide residues in the samples were quantitatively detected by gas chromatography-triple quadrupole mass spectrometry (GC-MS/MS) in the multiple reaction monitoring (MRM) mode. The 14 pesticide residues in the original ginsenoside extracts were effectively removed. The amounts of five residues were reduced to below 0.05 mg/kg, while the other nine residues were completely eliminated. This study demonstrates the application of HILIC to pesticide residue removal in traditional Chinese medicine extracts and reveals a new technique for the purification of natural products. The proposed method shows a high removal rate of pesticide residues and a high recovery of total ginsenosides. It is safe, efficient, and environment-friendly, and can aid the development of high-quality ginsenoside extracts.

人参(*Panax ginseng* C. A. Mey)为五加科植物人参的干燥根和根茎,具有大补元气,复脉固脱,补脾益肺,生津止渴,安神益智的功效^[1]^,是我国著名的名贵中药之一,被誉为“百草之王”^[2,3]^。人参提取物具有抗疲劳、抗衰老和保护心血管等诸多功效,被广泛应用于国内外保健品和膳食补充剂中,21世纪以来一直是我国出口美国最畅销的中药提取物之一^[4,5,6,7]^。而随着各个国家和地区对植物提取物中农药残留的监管日趋严格,农药残留超标问题已成为制约我国植物提取行业发展的重要因素,其中尤以人参提取物中的农残备受关注^[8,9,10,11,12]^。如美国食品药品管理局(food and drug administration, FDA)对人参提取物中农残检测多达500多项,且限量要求多为0.001~0.01 mg/kg,欧洲和日本虽然限量要求相对较为宽松,但检测项目也分别达到了400多项和200多项,极大地限制了我国人参提取物的出口^[8]^。

目前,常用的人参及人参制品中农药残留的脱除方法有有机溶剂法^[13,14]^、大孔树脂吸附法^[15,16,17]^和超临界萃取法^[18,19,20,21]^等。李广涛等^[22]^使用有机溶剂萃取法将人参提取物中腐霉利含量由44.51 mg/kg降至2.47 mg/kg,人参皂苷回收率为82.83%。该方法对腐霉利有一定的脱除效果,但未彻底脱除且人参皂苷损失严重;赵丽娟等^[23]^利用液液萃取和大孔吸附树脂法有效脱除人参茎叶中多种农药残留,但需使用氯仿、石油醚等有机溶剂,易对样品和环境造成污染;韩玉谦等^[24]^利用超临界1,1,1,2-四氟乙烷萃取技术可有效脱除人参中的有机氯农残,但易对样品造成二次污染且设备运行成本较高。

Alpert^[25]^在1990年首次提出亲水作用液相色谱(HILIC)的概念。HILIC主要采用极性固定相,水和有机溶剂为流动相,因其具有流动相组成简单、分离效率较高且与质谱有良好的兼容性等优势而越来越受到关注和重视,特别适用于分离强极性、带电荷的亲水化合物^[26,27]^。本实验根据农残和人参皂苷的结构特点,利用两类化合物间亲疏水性和相对分子质量的差异,在HILIC柱上分离人参皂苷和农残两类化合物,解决了现有脱除方法脱除不彻底、适用农残种类少、人参总皂苷损失率高和可能被二次污染等问题,并且安全高效、能较大程度地提高人参总皂苷纯度。

## 1 实验部分

### 1.1 仪器与试剂

Waters e2695型高效液相色谱仪(美国Waters公司), DAC50-动态轴向压缩柱(江苏汉邦科技有限公司), TSQ 8000 GC-MS/MS(美国Thermo Fisher Scientific公司), Milli-Q Integral 115超纯水系统(美国Millipore公司)

乙醇(食品级,泰州苏北化学试剂有限公司),乙酸乙酯、乙腈(质谱纯,德国默克公司),乙酸(分析纯,国药集团化学试剂有限公司),石墨化炭黑(艾览化工科技有限公司),十八烷基硅烷键合硅胶(C18)、*N*-丙基乙二胺(PSA)、硅胶、30 μm-C18YE、60 μm-Click XIon(华谱科仪科技有限公司),人参提取物(纯度 HPLC≥59.8%)、嘧菌酯(纯度≥99%)购自上海源叶生物科技有限公司, Rg_1_、Re、Rf、Rb_1_、Rc、Rb_2_、Rd纯度均≥98%,购自成都植标化纯生物技术有限公司,五氯硝基苯(纯度≥94%)、五氯苯胺(纯度≥96%)、丙环唑(纯度≥97%)、多菌灵(纯度≥97%)、异菌脲(纯度≥98%)、腐霉利(纯度≥98%)均购自阿拉丁试剂有限公司, 113种农药残留混合对照品购自天津阿尔塔科技有限公司。

### 1.2 仪器条件

色谱保留行为评价反相色谱 C18YE色谱柱(250 mm×4.6 mm, 10 μm);流动相为乙醇(A)和纯水(B)。洗脱程序为0~12 min, 0%A; 12~13 min, 0%A~60%A; 13~28 min, 60%A; 28~29 min, 60%A~90%A; 29~40 min, 90%A。柱温为30 ℃;检测波长为203 nm。

色谱保留行为评价亲水色谱 Click XIon色谱柱(250 mm×4. 6 mm, 5 μm);流动相为乙醇(A)和纯水(B)。洗脱程序为0~12 min, 95%A; 12~13 min, 95%A~60%A; 13~21 min, 60%A; 21~22 min, 60%A~40%A; 22~28 min, 40%A。流速为1.0 mL/min;柱温为30 ℃;检测波长为203 nm。

皂苷样品液相色谱分析 Click XIon色谱柱(250 mm×4.6 mm, 5 μm);流动相为乙腈(A)和纯水(B)。洗脱程序为0~20 min, 90%A~60%A。流速为1.0 mL/min;柱温为30 ℃;进样量为10 μL;检测波长为203 nm。

亲水色谱制备 DAC50-动态轴向压缩柱,UV检测器,检测波长为203 nm, Click XIon色谱填料(填料重量300 g,粒径60 μm),流速60 mL/min,以95%乙醇溶液为溶剂,将30 g人参提取物样品溶解为600 mL,采用泵上样方式,以乙醇(A)和纯水(B)作为流动相,洗脱程序为0~10 min,上样;10~20 min, 95%A淋洗;20~30 min, 90%A洗脱得到溶液Ⅰ; 30~45 min, 60%A洗脱得到溶液Ⅱ, 45~55 min, 40%A洗脱得到溶液Ⅲ。

气相色谱 色谱柱为Thermo农残专用柱(30 m×0.35 mm, 0.25 μm);载气为高纯氦气,流速1.2 mL/min;进样口温度270 ℃;色谱柱升温程序为:40 ℃保持1.5 min,以25 ℃/min升至90 ℃,保持1.5 min,再以25 ℃/min升至180 ℃,保持1.5 min,以5 ℃/min升至280 ℃,最后以10 ℃/min升温到300 ℃,保持5 min;进样方式为不分流进样;进样量为1 μL。

质谱 电子轰击源70 eV,离子源温度300 ℃,传输线温度280 ℃;数据采集方式为多反应监测(MRM)模式。

### 1.3 标准溶液的配制

准确吸取1 mL质量浓度为10 mg/L的113种农药残留混合对照品于10 mL容量瓶中,加入乙腈定容,得到质量浓度为1 mg/L的混合标准储备溶液。使用乙腈稀释对照品溶液,配制成质量浓度为5、20、100、300、600、1000 μg/L的混合对照品溶液,储存于-18 ℃备用。

### 1.4 供试品溶液的配制

工艺条件优化中的供试品溶液 精密称定人参提取物0.250 g 3份,分别置于5 mL、10 mL、10 mL 3个容量瓶中,在1个5 mL和1个10 mL的容量瓶中,分别加入五氯硝基苯、五氯苯胺、腐霉利各1 mg,然后加入95%乙醇充分溶解,定容,摇匀,得到未添加农药标样的25 g/L人参提取物供试品溶液,添加农药标样的25 g/L人参提取物供试品溶液和添加农药标样的50 g/L人参提取物供试品溶液,分别标记为原液Ⅰ、Ⅱ、Ⅲ。

农药残留检测中的供试品溶液 参照2015版《中国药典》第四部多种农药残留量的测定中供试品溶液的制备方法制备^[28]^。

### 1.5 定量及纯度计算方法

HPLC分析含农残人参提取物试样时,农残脱除率和人参总皂苷(以Rg_1_、Re、Rf、Rb_1_、Rc、Rb_2_、Rd 7种皂苷计算,下同)回收率计算公式如下:


(1) $回收率(脱除率)=\frac{A_{i}V_{i}}{A_{x}V_{x}}\times 100\%$

式中:*A_i_*为所分析馏分中人参皂苷(农残)的总峰面积,*V_i_*为所分析馏分的体积;*A_x_*为原液分析时农残或人参皂苷的总峰面积,*V_x_*为原液的上样体积。

根据体积和峰面积计算人参总皂苷样品纯度,公式如下:


(2) $纯度=\frac{A_{n}}{A_{m}}\times 100\%$

式中:*A_n_*为所分析样品溶液中人参皂苷的总峰面积;*A_m_*为所分析样品溶液中所有成分的总峰面积。

根据上样时原液中人参提取物的质量浓度、上样体积及色谱柱中填料质量计算上样量,公式如下:


(3) $上样量=\frac{C_{a}V_{a}}{m_{b}}\times 100\%$

式中:*C*_a_为原液中人参提取物的质量浓度;*V*_a_为原液的上样体积;*m*_b_为色谱柱中填料质量。

## 2 结果与讨论

### 2.1 农药与人参总皂苷色谱保留行为评价

基于疏水保留原理的大孔树脂是目前工业上用于植物提取物精制,提高有效成分含量的主要工艺手段^[29,30]^。在树脂精制过程中也可以同时去除大量的农药残留,但仍有部分农药会与有效成分共流出,并在精制过程中一起被富集,从而在最终的产品中严重超标。本实验以人参提取物中易超标的7种农药标准品和市售总皂苷样品为例,对比了其在反相色谱填料和亲水色谱填料上的保留行为差异。为适应后续工业生产工艺的开发,模拟制备液相色谱方法,采用不同比例的乙醇水体系进行等度洗脱。结果如图1所示:在反相色谱中,除五氯硝基苯和五氯苯胺保留较强,能与皂苷类成分显著分离外,多菌灵、嘧菌酯、腐霉利、异菌脲和丙环唑5种农药都与人参总皂苷的保留相近;而在亲水色谱中,与总皂苷成分保留情况不同,7种农药在95%乙醇条件下仅有微弱保留,与总皂苷样品实现了良好的分离。Click XIon固定相典型的表面化学结构和典型人参皂苷及7种农药的结构式如图2所示。从农药与总皂苷成分在反相色谱柱上的保留规律来看,基于类似的疏水保留机理,树脂纯化后的总皂苷样品,即使采用分离柱效更高的球形硅胶基质的反相填料也很难将农残进一步脱除。而在Click XIon亲水色谱中,由于固定相表面同时具有带正负电荷的官能团,使其具有较强的亲水性^[27]^。根据之前文献报道,皂苷糖基上的羟基可与Click XIon固定相表面的羧基形成氢键,而这种氢键作用是皂苷在Click XIon柱上保留的主要次级作用,使得总皂苷样品因为糖链的存在而具有较强的亲水保留^[31]^,而这7种农药由于相对分子质量较小,亲水性较差,在亲水色谱填料上保留微弱。正是基于两者保留行为上的巨大差异,本实验拟开发一种适用于人参提取物中农残脱除的方法。

**图 1:**
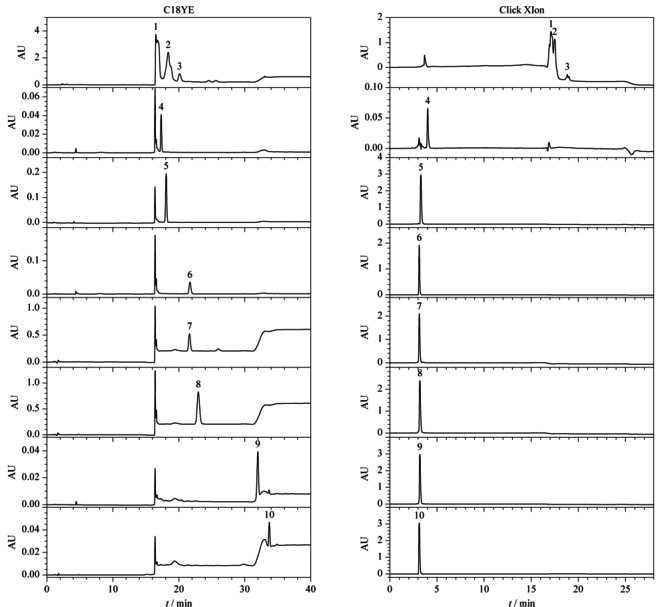
7种农药与人参总皂苷标准品在反相和亲水色谱柱上的色谱图

**图 2:**
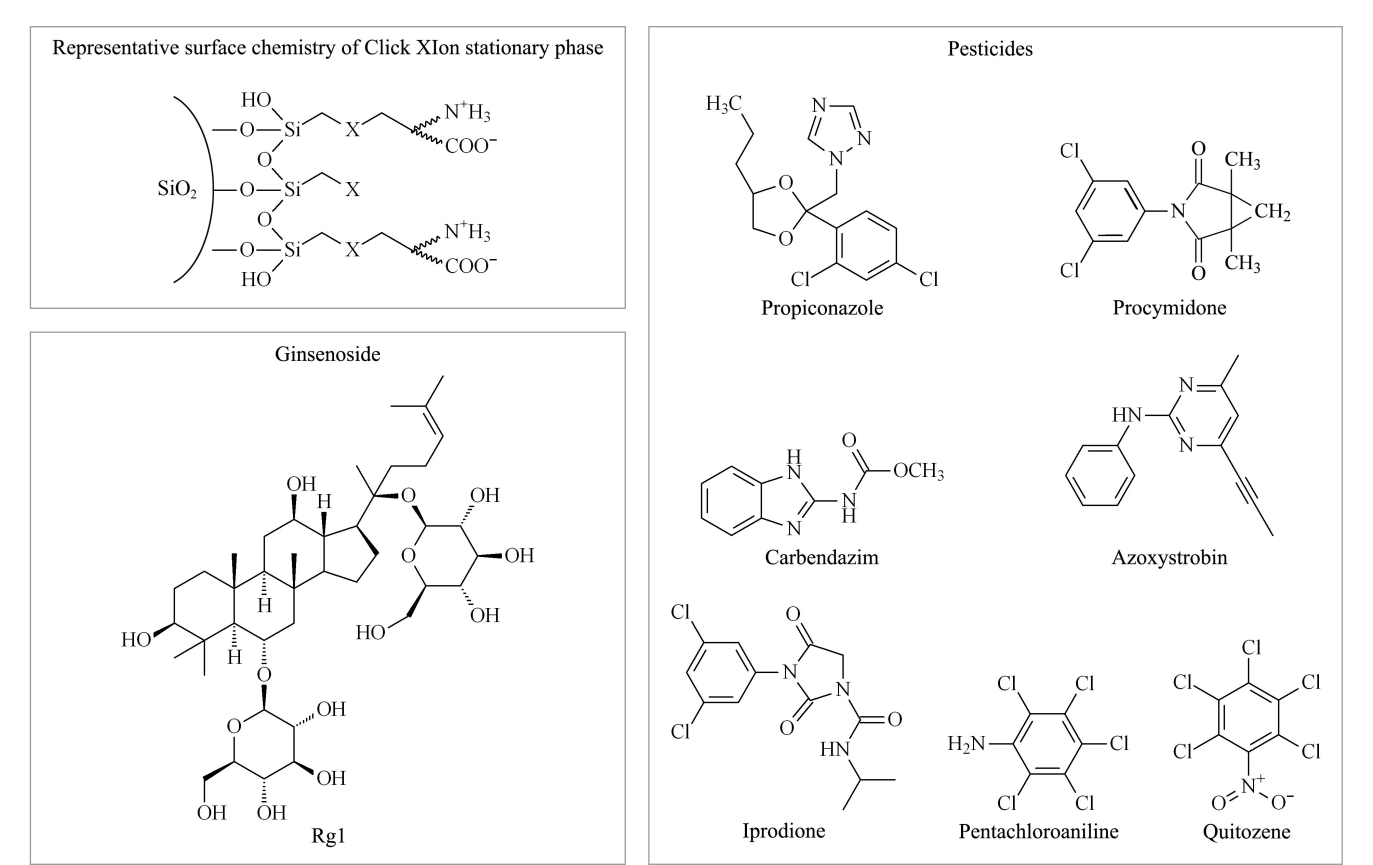
Click XIon固定相典型的表面化学结构和典型人参皂苷及农药结构式

### 2.2 农残脱除工艺条件优化

使用装有2 g Click XIon填料(粒径60 μm)的SPE柱,对该亲水填料脱除农残的最大上样量、淋洗体积和上样体积进行考察。

2.2.1 最大上样量考察

为减少实验过程中皂苷损失,对最大上样量进行考察。将上述1.4节中原液Ⅰ上样,并对上样流出液进行即时检测,每上样1BV(为便于计算,统一以填料重量对应的数值为1BV,如2 g填料的1BV定义为2 mL, 300 g填料的1BV定义为300 mL,下同)即对其相对应的流出液进行液相色谱分析,如图3中b、c、d和e即分别为上样流出液Ⅰ、Ⅱ、Ⅲ和Ⅳ分析所得色谱图。图3a、f、g、h分别对应的溶液为原液Ⅰ、95%乙醇溶液淋洗所得淋洗液、80%乙醇溶液洗脱所得洗脱溶液Ⅰ和60%乙醇溶液洗脱所得洗脱溶液Ⅱ。如图3e中所示,当上样流出第4BV,即上样4BV,根据公式(3)计算上样量为10%时,上样流出液中出现皂苷峰,根据峰面积计算,在所上样皂苷中的含量占比为1.1%,说明该上样量已接近填料载样的极限,故选择10%的上样量做进一步的条件优化。

**图 3:**
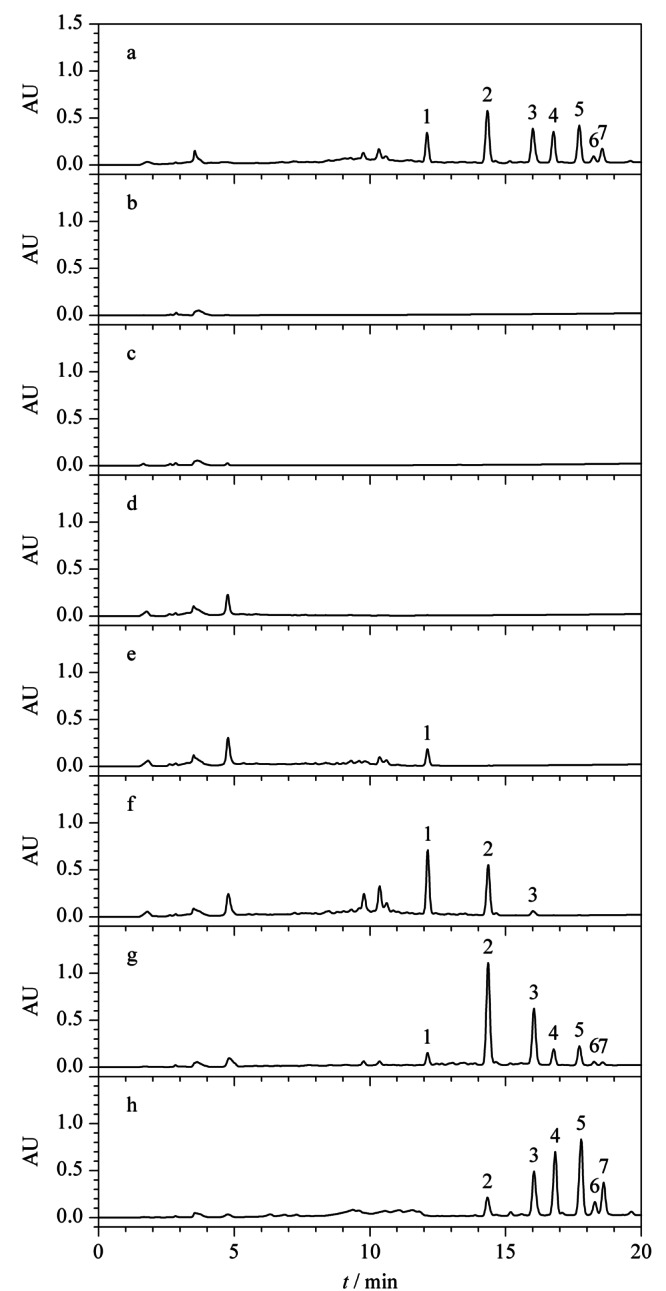
最大上样量考察时不同馏分的分析色谱图

2.2.2 淋洗体积考察

根据上述2.2.1中结果,将原液Ⅱ以10%总皂苷的上样量上样,绝大部分目标化合物保留在SPE柱上。如图4中a为原液Ⅱ分析所得色谱图,b为原液经过SPE柱后流出的溶液分析所得色谱图,从图4可知,在10%上样量下,农药和人参总皂苷均有部分流穿。为将仍吸附在填料上的农药冲洗下来,同时保证人参总皂苷不被洗脱,以95%乙醇溶液为淋洗液,以1 mL即0.5BV为单位对淋洗液体积进行依次分析,考察最佳淋洗体积。图4中c、d、e、f为对应的淋洗液Ⅰ、Ⅱ、Ⅲ和Ⅳ分析所得色谱图,从图e中可明显看出,当淋洗体积为1.5 BV时开始有人参皂苷流出,继续淋洗1 mL后,人参总皂苷损失加大,农残峰的峰面积减小。当淋洗体积为2 BV时,从图4g和h中可看出,洗脱馏分色谱图中无明显农残峰存在,此时根据公式(1)计算,农残总脱除率为101.8%,上样过程中人参总皂苷损失率为3.5%,淋洗过程中人参总皂苷损失率为3.9%。根据农残脱除结果,选择淋洗体积为2 BV。

**图 4:**
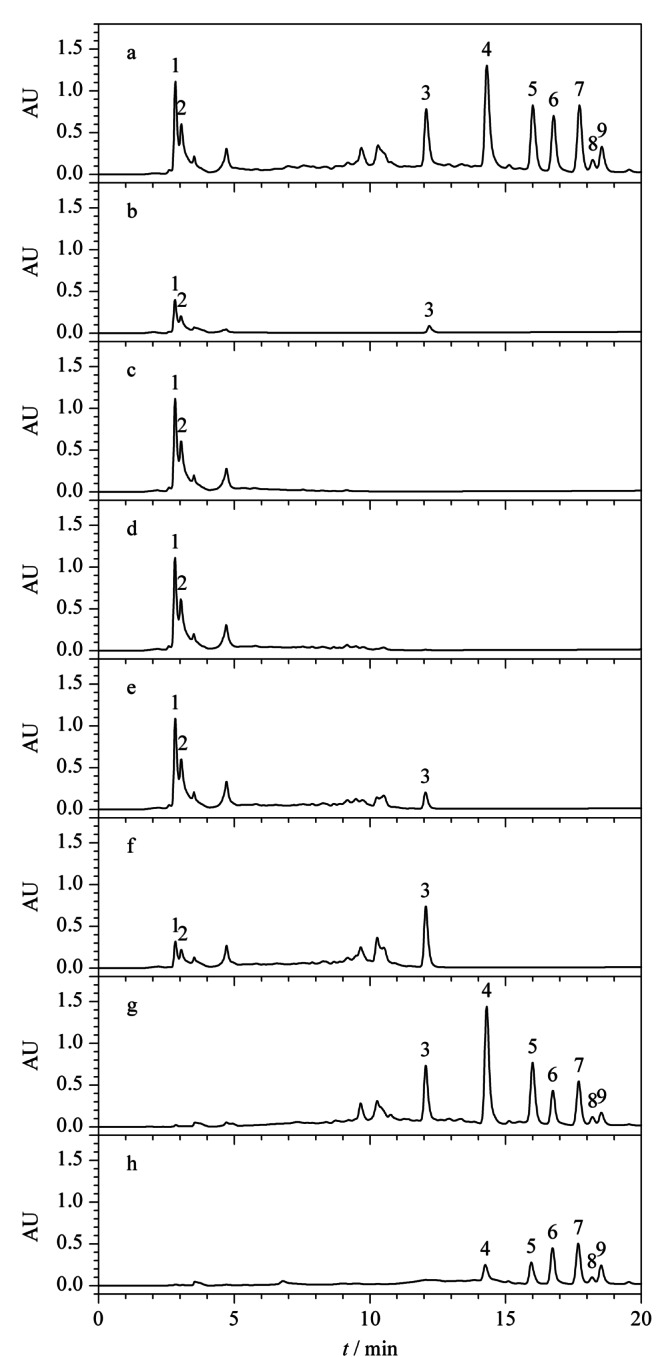
淋洗体积考察时不同馏分的分析色谱图

2.2.3 上样体积考察

在上述2.2.1和2.2.2节中的上样浓度和体积下,存在部分人参总皂苷穿透现象,可能是由于上样体积太大造成,故而将上样液浓度增大,上样体积减小进行SPE小试试验。如图5中a、b分别为原液Ⅲ和上样流出液分析所得色谱图,从图中可明显看出,在所确定的最大上样量和淋洗体积条件下,将上样体积减小至2 BV,上样流出液中未出现人参皂苷峰。如图5c为95%乙醇溶液淋洗所得淋洗液分析所得色谱图,从图中可看出人参总皂苷有少量流出损失,经计算,人参总皂苷损失率由7.4%减小至3.4%,且减少了溶剂的使用,提高了效率,在实际工艺生产中,会降低生产成本。从洗脱馏分色谱图5d和e可看出,无明显农药峰存在,根据峰面积计算农药总脱除率为102.1%,说明减少上样体积在保证农残脱除率情况下,能降低人参总皂苷损失,故最终确定上样体积为2 BV。

**图 5:**
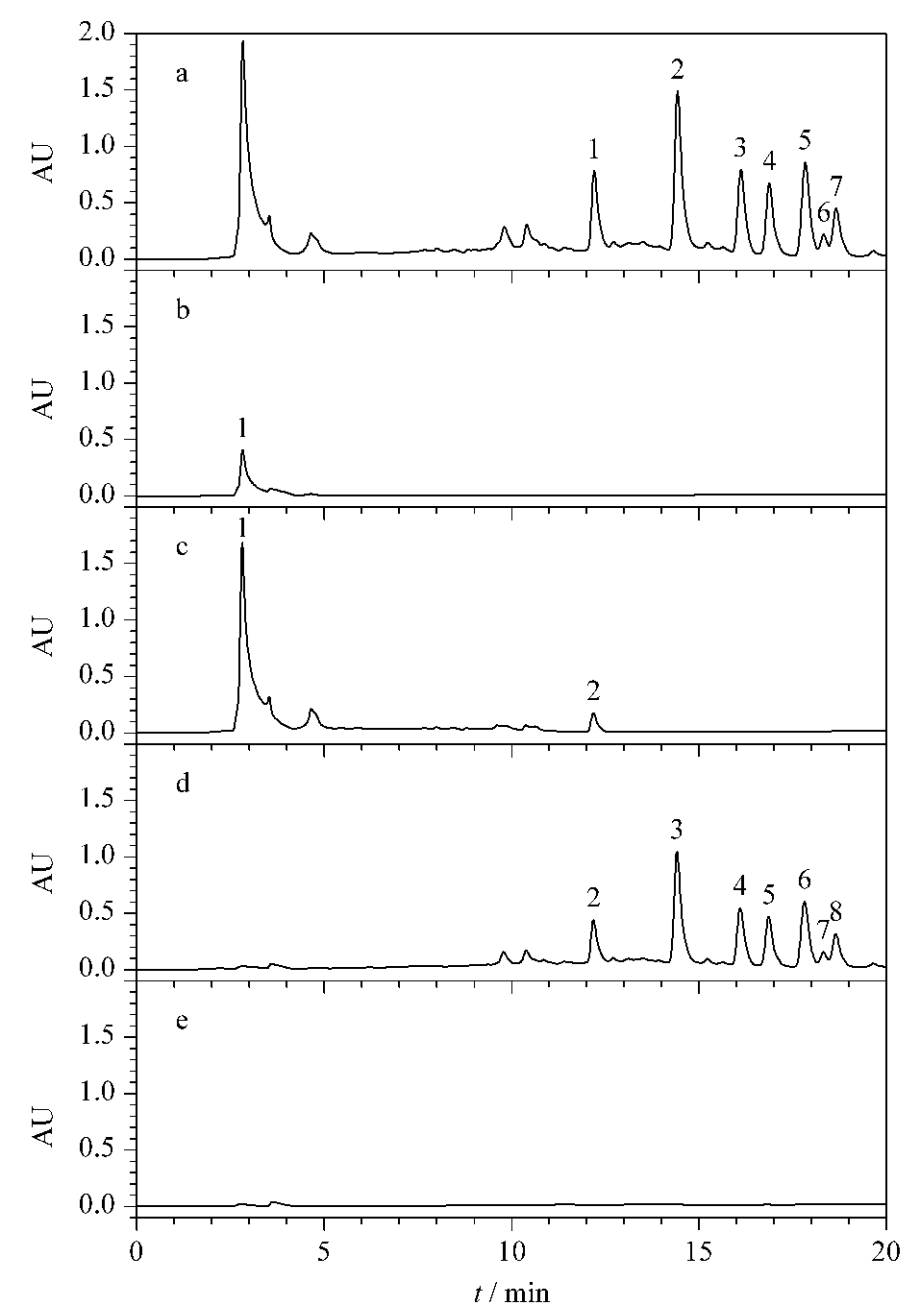
上样体积优化时不同馏分的分析色谱图

### 2.3 实际样品制备

根据所优化的最佳工艺条件,在DAC50系统上开展放大制备实验,具体条件见上述1.2节,制备谱图如图6所示。对制备所得各馏分进行液相色谱分析,根据公式(1)计算,具体各馏分中人参总皂苷含量占比如表1所示,合并洗脱液Ⅰ和Ⅱ,根据公式(2)计算样品纯度,最终人参总皂苷回收率为94.4%,且纯度由59.87%提高到69.61%。

**图 6:**
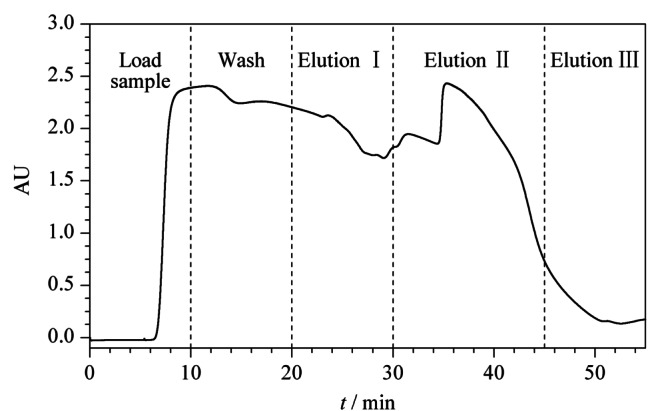
人参总皂苷的制备色谱图

**表 1:** 各馏分中人参总皂苷峰面积的百分比及成品纯度

Fraction/ min	Category	Volume/ mL	Ginsenosides		
			Peak area	Percentage/ %	Purity/ %
0-10	flow-through	600	1319	0.02	-
	solution				
10-20	wash solution	600	531596	7.22	-
20-30	eluent Ⅰ	600	1627184	22.10	-
30-45	eluent Ⅱ	900	3565080	72.63	-
45-55	eluent Ⅲ	600	2734	0.04	-
-	stock solution	600	7362825	-	59.87
-	combined	1500	2945130	94.4	69.61
	solution				

Combined solution: combine eluents Ⅰ and Ⅱ.

采用GC-MS/MS对亲水色谱脱除前后人参总皂苷样品进行113项农残检测,参考赛默飞世尔科技对植物性食品中208种农残检测方法包^[32]^,确定113种农药保留时间及定性和定量离子对,通过对上述1.3节中所配制的混合对照品溶液进行分析,分别绘制出113种农药的标准曲线,所得113种农药的线性相关系数(*R*^2^)均大于0.99,通过线性回归方程对其进行定量计算。检测出脱除前样品中含有14种含量达0.1 mg/kg及以上农残,其中皮蝇磷、克百威和甲基毒死蜱含量在0.5 mg/kg以上,经亲水色谱脱除后,其中9种农药未检出,另外5种含量降至0.05 mg/kg以下。14种农药脱除前后具体含量见表2,结果证实亲水色谱对人参总皂苷中农药残留具有良好的脱除效果。

**表 2:** 精制样品中含量在0.1 mg/kg及以上的14种农残脱除前后含量对比

No.	Retention time / min	Analyte	Contents/(mg/kg)	
			Before removal	After removal
1	11.67	fenobucarb (仲丁威)	0.141	0.03
2	12.81	phorate (甲拌磷)	0.116	0.02
3	13.13	atraton (阿特拉通)	0.137	-
4	13.36	carbofuran (克百威)	0.663	-
5	14.17	pyrimethanil (嘧霉胺)	0.184	-
6	15.43	chlorpyrifos-methyl	2.113	-
		(甲基毒死蜱)		
7	15.88	ronnel (皮蝇磷)	0.513	-
8	16.28	ethofumesate (乙氧呋草黄)	0.199	0.03
9	17.13	isocarbophos (水胺硫磷)	0.103	-
10	18.53	procymidone (腐霉利)	0.360	-
11	19.11	tetrachlorvinphos (杀虫畏)	0.316	-
12	19.73	isoprothiolane (稻瘟灵)	0.114	0.03
13	22.70/	propiconazole (丙环唑)	0.164	-
	22.87			
14	24.48	phosmet (亚胺硫磷)	0.233	0.04

-: not detected (content is less than 0.01 mg/kg).

## 3 结论

本文以人参提取物为研究对象,利用人参皂苷与农残之间相对分子质量和亲疏水性的差异,考察了人参中常见农残和人参总皂苷在反相和亲水色谱柱上的保留行为,确定了亲水色谱脱除人参提取物中农药残留的最佳上样量和洗脱条件,并放大到制备水平,人参总皂苷回收率达94%以上且纯度提高近10%,对脱除前后样品进行113项农残检测,亲水色谱脱除前样品中含量达0.1 mg/kg及以上农残共14种,经亲水色谱脱除,所得成品中113种农残均未检出或含量在0.05 mg/kg以下。与现有的农残脱除方法相比,该方法简单、高效、稳定,且适用于多种农残脱除,为高品质人参及其他中药提取物产品的开发及生产提供参考。
